# Editorial: Innovative prevention strategies for occupational health hazards

**DOI:** 10.3389/fpubh.2025.1584770

**Published:** 2025-03-18

**Authors:** Chun-Yip Hon, Enrico Bergamaschi

**Affiliations:** ^1^School of Occupational and Public Health, Toronto Metropolitan University, Toronto, ON, Canada; ^2^Department of Public Health and Pediatric Sciences, University of Turin, Turin, Italy

**Keywords:** editorial, occupational health hazards, prevention, innovative, health and safety (H&S)

Health hazards are abound in workplaces across the globe. In fact, according to the International Labor Organization, approximately three million people die annually as a result of work-related accidents and disease (1). Besides the death of workers along with their pain and suffering, the cost of workplace accidents and illnesses worldwide is estimated to be almost 3 trillion dollars (2). Any reasonable person will argue that these numbers are disturbingly high and unacceptable. A collective effort by government, workplaces, worker advocacy groups, and occupational health and safety professionals, to name a few, is required to lower these dire statistics. We recognize the importance of taking advantage of ways to reduce the risk of occupational health hazards adopted by different organizations worldwide. This is noteworthy as the intention of the Research Topic was to highlight *Innovative prevention strategies for occupational health hazards*. Fourteen articles were published on various Research Topics including chemical hazards, thermal stress, psychosocial hazards as well as ergonomics from multiple countries. Below is a summary of these articles that were published in the Research Topic (in chronological order).

According to the World Health Organization (WHO), “burnout” results from chronic unmanageable workplace stress (3). In their 2023 Work in America Survey, the American Psychological Association reported that 57% of workers experienced negative impacts due to work-related stress that are associated with burnout such as emotional exhaustion and lack of motivation (4). In the study by Aminihajibashi et al., the research team examined burnout and secondary traumatic stress in child mental health clinicians—an under-researched occupational cohort. The authors suggest that interventions to preventing burnout include improved organizational climate and work-life balance.

Musculoskeletal disorders (MSDs) amongst nurses has been a known concern for quite some time and the prevalence of MSD in this cohort, specifically low back pain, is estimated to be as high as 80% (5). In the article by Rakhshani et al., they adopted the PRECEDE-PROCEED model, a health promotion framework, toward an education intervention which led to the improvement of knowledge, attitudes and preventative behaviors with respect to MSDs amongst a group of nurses.

In their mini review, Tang et al. hints at the potential for metaverse technology, a virtual world that is fully immersive and interactive (6), to be employed toward health and safety training, risk identification and assessment, as well as occupational disease monitoring and diagnosis.

Due to using monitors and other devices on a regular basis, computer vision syndrome (CVS) may result. CVS is a set of ocular symptoms including eye strain, redness, irritation, tired eyes, double vision and blurred vision (7). Gasheya et al.'s study aimed to determine the prevalence of CVS among commercial bank workers in Ethiopia and identify ergonomic risk factors. Overall, poor ergonomics setup of an office workstation and glare were associated with increased CVS—both of which are novel findings and should be taken into consideration to reduce the likelihood of CVS.

Shoker et al. conducted a systematic review of randomized-control trials assessing the effect of mindfulness programs on burnout. Two-thirds of the trials examined demonstrated that these mindfulness programs had a significant beneficial effect on burnout metrics, with the most impacted element being emotional exhaustion.

Risk assessment is a fundamental principle of occupational health and safety and is intended to identify, assess, and control hazards in the workplace and, in turn, improve the health and safety of workers (8). However, there is no standardized approach for conducting a risk assessment of occupational hazards, especially in small and micro enterprises. Hollá et al. developed and validated a new systematic risk assessment procedure with positive outcomes that can be employed for a variety of occupations and procedures/tasks.

Exposure to nanomaterials is a growing occupational health concern made more complicated by the fact that there is no standardized exposure assessment method as well as no published occupational exposure limits (9). One possible means to reduce the risk of exposure to nanoparticles generated from 3D printing is employing safe(r)-by-design (SbD) principles. In their research article, McLean et al. collected airborne samples of nanomaterials from 3D printing processes in order to populate an exposure database used to support decision-making of the European Standard for monitoring release of nanomaterials as well as to facilitate the application of SbD strategies toward reducing occupational exposure to these airborne hazards.

An estimated 15% of working-age adults have a mental disorder according to the WHO (10). Further, the WHO estimates that 12 billion working days are lost annually due to depression and anxiety which results in lost productivity costing US$ 1 trillion per year (10). To address this, a framework to create mentally healthy workplaces was originally released in 2018 (11). The same group of researchers, led by Deady, updated the framework in their article published in this Research Topic. This update incorporated evidence-based best practices as well as other findings from new research regarding the effectiveness of interventions which are aimed to foster mentally healthy workplaces.

Workplace health promotion programs have been deemed as being important to modify behavior and risk factors associated with various outcomes including weight-related issues, mental health, and musculoskeletal disorders (12). In their study, Hente and Schlesinger conducted a qualitative study to assess the perception of organizations with respect to a workplace health management network (WHM) aimed at health promotion in small and medium enterprises. They concluded that the WHM network was beneficial for increasing awareness and implementation of health-promoting interventions.

The frequency and intensity of wildfires is increasing (13). This is an issue as wildland firefighters are already at risk of heat stress (14) and it has been shown that their core body temperature rises when the amount of physical exertion increases (i.e., fighting a more intense fire) (15). Gutiérrez-Arroyo et al.'s study examined the effectiveness of two cooling interventions, a cooling vest and removal of personal protective equipment, in simulated environments mimicking wildland firefighting conditions. Unfortunately, neither cooling strategy was found to effectively mitigate thermal strain amongst wildland firefighters.

n-Hexane is a chemical widely used in various production processes including printing. Occupational exposure to n-hexane can lead to damage to the peripheral nerves and such cases have been reported in countries throughout the world (16). Given its frequency of use and associated health effects, Hu et al. sought to analyze n-hexane exposure in the printing industry in China using various occupational health risk assessment models. They concluded that the method outlined in China's Technical Guide GBZ/T 289-2017 was most appropriate for assessing exposure risk to n-hexane in printing enterprises.

In the third and final burnout study published in the Research Topic, Fang et al. examined if a relationship exists between humor styles and burnout in nurses. Their findings suggest that using humor to cope with stress and maintain a positive attitude may be beneficial in mitigating burnout among nurses.

Utilizing computer vision, which automatically extracts meaningful information from images, has the potential to improve workplace health and safety (17). Román-Gallego et al. used a form of artificial neural network to recognize safety signs—regardless of their orientation. The findings suggest that the artificial neural network is capable of recognizing the signage and, in turn, alerting workers of risks. However, errors did occur, and further research is needed.

Psychosocial hazards in the workplace can lead to various adverse health effects including mental health issues, musculoskeletal disorders and cardiovascular disease (18). Moreover, these hazards can have negative effects on workers' job satisfaction as well as their productivity (19). In their study, Bazaluk et al. developed a method for determining psychosocial risk factors that aligns with ISO 45003:2021 (Occupational health and safety management: Psychological health and safety at work—Guidelines for managing psychosocial risks) (20). The authors developed a systematic approach to assess psychosocial risk factors which, in turn, can be used to manage these same risks.

Overall, this Research Topic showcases some novel approaches to address various occupational health hazards. It is hoped that organizations will consider adopting some of these measures to improve health and safety in the workplace. Given the startling statistics presented earlier, any opportunity to reduce the frequency and severity of workplace exposures/incidents should be contemplated as the health and wellbeing of literally millions of people is at stake.
